# Publisher Correction: Nfat/calcineurin signaling promotes oligodendrocyte differentiation and myelination by transcription factor network tuning

**DOI:** 10.1038/s41467-018-03871-z

**Published:** 2018-04-23

**Authors:** Matthias Weider, Laura Julia Starost, Katharina Groll, Melanie Küspert, Elisabeth Sock, Miriam Wedel, Franziska Fröb, Christian Schmitt, Tina Baroti, Anna C. Hartwig, Simone Hillgärtner, Sandra Piefke, Tanja Fadler, Marc Ehrlich, Corinna Ehlert, Martin Stehling, Stefanie Albrecht, Ammar Jabali, Hans R. Schöler, Jürgen Winkler, Tanja Kuhlmann, Michael Wegner

**Affiliations:** 1 Institut für Biochemie, Emil-Fischer-Zentrum, Friedrich-Alexander-Universität Erlangen-Nürnberg, D-91054 Erlangen, Germany; 2 Institute of Neuropathology, University Hospital Münster, D-48149 Münster, Germany; 3 Department of Cell and Developmental Biology, Max Planck Institute for Molecular Biomedicine, D-48149 Münster, Germany; 4 Department of Molecular Neurology, University Hospital Erlangen, Friedrich-Alexander-Universität Erlangen-Nürnberg, D-91054 Erlangen, Germany

Correction to: *Nature Communications* 10.1038/s41467-018-03336-3; published online 02 March 2018

The originally published version of this Article omitted Tanja Kuhlmann and Michael Wegner as jointly supervising authors. This has now been corrected in both the PDF and HTML versions of the Article.

