# Supplementary material for: Nfat/calcineurin signaling promotes oligodendrocyte differentiation and myelination by transcription factor network tuning
Source: Nat Commun. 2018 Mar 2;9:899. doi: 10.1038/s41467-018-03336-3 (PMC5834605; doi:10.1038/s41467-018-03336-3)
Supplement: Supplementary file 1 — Supplemtary Information [file 41467_2018_3336_MOESM1_ESM.pdf]

Supplementary Information

for

Nfat/calcineurin signaling promotes oligodendrocyte differentiation and myelination by transcription factor network tuning

by

Matthias Weider, Laura Julia Starost, Katharina Groll et al.

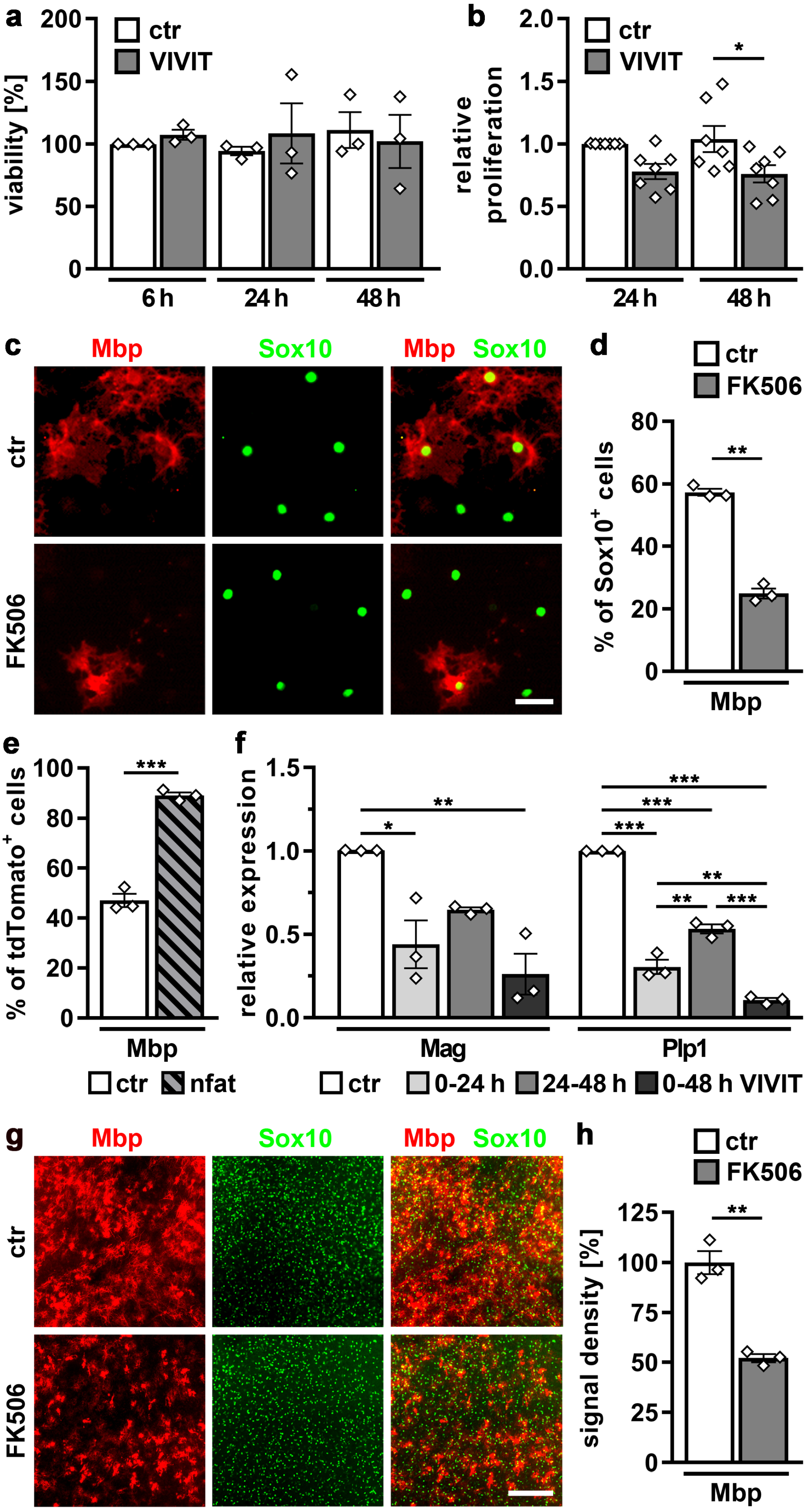

a
viability [%]
ctr
VIVIT
6 h
24 h
48 h
b
relative proliferation
24 h
48 h
c
Mbp
Sox10
Mbp Sox10
ctr
FK506
d
% of Sox10+ cells
Mbp
e
% of tdTomato+ cells
Mbp
ctr
nfat
f
relative expression
Mag
Plp1
ctr
0-24 h
24-48 h
0-48 h VIVIT
g
Mbp
Sox10
Mbp Sox10
ctr
FK506
h
signal density [%]
Mbp

Supplementary Figure 1: Nfat/calcineurin signaling is required for oligodendroglial differentiation in culture. (a) Viability of mouse oligodendroglial cells kept for 6 h, 24 h and 48 h under proliferating conditions in culture in the absence (ctr, open bars) or presence (grey bars) of 1 µM VIVIT (n = 3). Viability after 6 h in the absence of VIVIT was arbitrarily set to 100 percent and used to normalize in pairwise fashion (values: 100 ± 0% for control and 108 ± 4% for VIVIT treatment after 6 h; 95 ± 3% for control and 109 ± 24% for VIVIT treatment after 24 h; 111 ± 14% for control and 102 ± 21% for VIVIT treatment after 48 h). (**b**) In these cells, BrdU incorporation was also determined (n = 7). The number of proliferating cells after 24 h under control conditions was arbitrarily set to 1 and used to normalize in pairwise fashion (values: 1 for 24 h under control conditions, 0.92 ± 0.05 for 24 h VIVIT treatment, 0.82 ± 0.02 for 48 h under control conditions, 0.76 ± 0.13 for 48 h VIVIT treatment). **(c,d)** Immunohistochemical staining (c) and corresponding quantifications (n = 3) (d) of rat oligodendroglial cells kept for 4 days in culture under differentiating conditions in the absence (ctr) or presence of 1 µM FK506. Antibodies directed against Sox10 (green) and Mbp (red) were used. Scale bar, 20 µm. The number of Sox10-positive cells in each culture was set to 100% (Mbp values: Mbp 57.3 ± 1.1 in control cultures and 24.9 ± 1.6 in FK506-treated cultures). (**e**) Quantification of cells co-expressing Mbp and tdTomato in rat oligodendroglial cultures. Constructs carrying a tdTomato reporter under control of a minimal promoter (ctr, open bar) or minimal promoter and multimerized Nfat binding sites (nfat, hatched bar) were introduced into cells by retroviral transduction one day prior to differentiation. After 6 days of differentiation the percentage of tdTomato-expressing cells with additional Mbp expression was determined (n = 3; values: 47.1 ± 4.6 for ctr and 89.1 ± 2.1 for nfat). (**f**) Analysis of *Mag* and *Plp1* expression in mouse oligodendroglial cells cultured for 48 h under differentiating conditions in the absence (ctr, open bar) or presence of 1 µM VIVIT (grey bars) (n = 3). Incubation with VIVIT was restricted to the first 24 h (light grey bars) or the second 24 h (grey bars) of incubation or throughout the whole cultivation period (dark grey bars). Cultures were used to prepare RNA and perform qrtPCR. The amount of *Mag* and *Plp1* transcripts present after 48 h under control conditions was arbitrarily set to 1 and used to normalize (*Mag* values: 1 for control conditions, 0.44 ± 0.14 for VIVIT treatment during the first 24 h, 0.65 ± 0.01 for VIVIT treatment during the second 24 h, 0.26 ± 0.12 for 48 h VIVIT treatment; *Plp1* values: 1 for control conditions, 0.31 ± 0.04 for VIVIT treatment during the first 24 h, 0.53 ± 0.03 for VIVIT treatment during the second 24 h, 0.11 ± 0.01 for 48 h VIVIT treatment). **(g)** Immunohistochemistry of cortical slices from newborn mice with anti-MBP (red) and anti-Sox10 (green) antibodies in the absence (ctr) and presence of 1 µM FK506 after 7 days in culture. Scale bar, 200 µm. **(h)** Quantification of Mbp signal density in FK506-treated and control cortical slices (n = 3; values: 100 ± 5.8 for control and 52.1 ± 2.0 for FK506 treatment). Statistical significance was determined by Bonferroni-corrected two-way ANOVA in (a) and (b), Bonferroni-corrected one-way ANOVA in (f) and two-tailed Student's t-test in (d), (e) and (h) (*, P ≤0.05; **, P ≤0.01; ***, P ≤0.001).

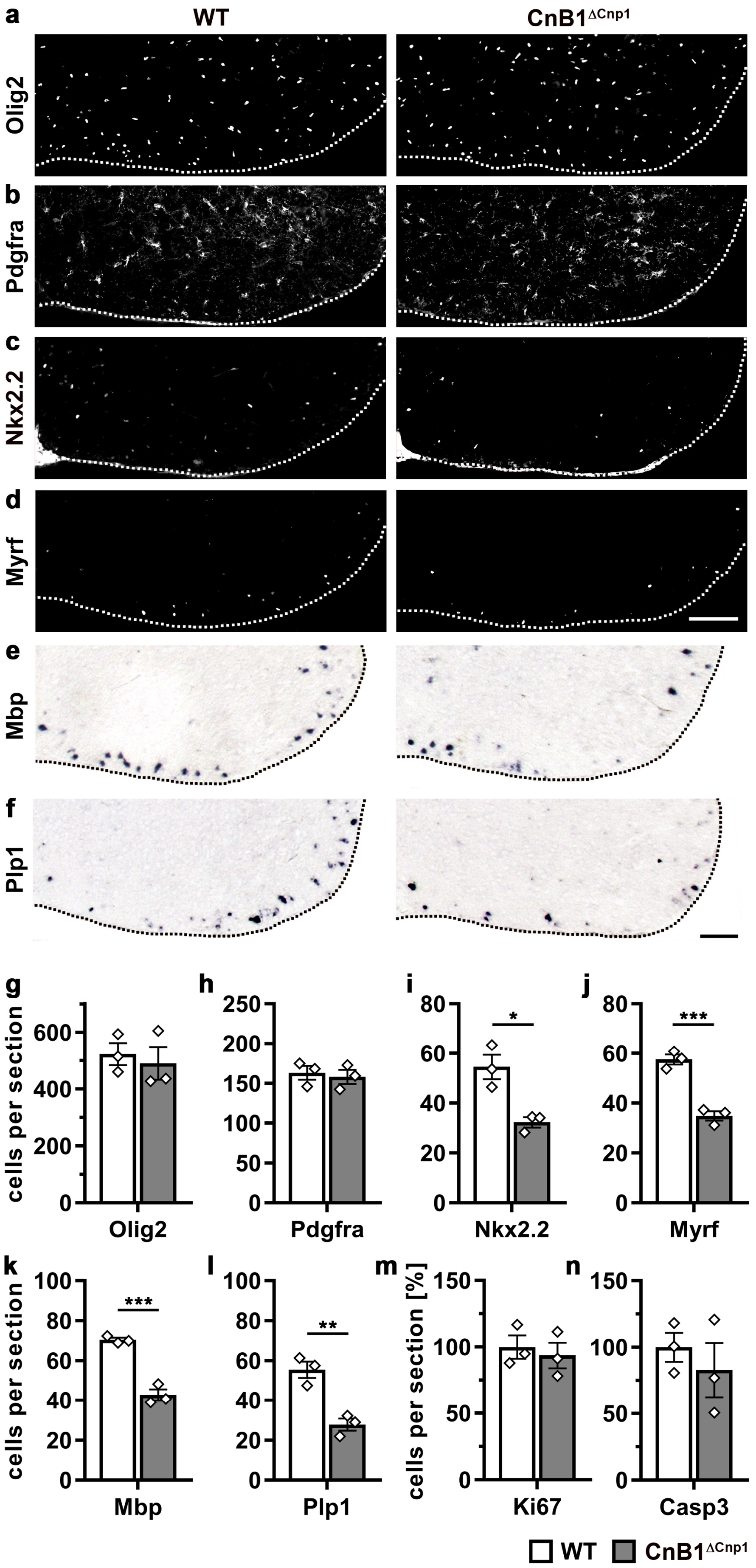
a
WT
CnB1$^{\Delta Cnp1}$
Olig2
b
Pdgfra
c
Nkx2.2
d
Myrf
e
Mbp
f
Plp1
g
h
i
j
cells per section
Olig2
Pdgfra
Nkx2.2
Myrf
*
***
k
l
m
n
cells per section
cells per section [%]
Mbp
Plp1
Ki67
Casp3
***
**
WT
CnB1$^{\Delta Cnp1}$

Supplementary Figure 2: Nfat/calcineurin signaling is not required in oligodendroglial cells before differentiation (a-f) Analysis of oligodendroglial marker expression in 18.5 dpc-old embryos of wildtype (WT) and $CnB1^{\Delta Cnp1}$ mice by immunohistochemistry and in situ hybridization. Antibodies were directed against Olig2 (a), Pdgfra (b), Nkx2.2 (c), and Myrf (d). Riboprobes recognized *Mbp* (e) and *Plp1* (f) mRNA. Scale bars, 100 µm. (**g-n**) From these and similar stainings for Ki67 (m) and cleaved caspase 3 (n) quantifications were performed on three embryos for each genotype (n = 3) counting three separate sections. Presentations are as absolute (g-l) or relative (m,n) numbers (values: Olig2: 523 ± 39 for WT and 490 ± 58 for $CnB1^{\Delta Cnp1}$; Pdgfra: 163 ± 9 for WT and 158 ± 9 for $CnB1^{\Delta Cnp1}$; Nkx2.2: 55 ± 5 for WT and 32 ± 2 for $CnB1^{\Delta Cnp1}$; Myrf: 58 ± 2 for WT and 35 ± 2 for $CnB1^{\Delta Cnp1}$; *Mbp*: 70 ± 2 for WT and 43 ± 3 for $CnB1^{\Delta Cnp1}$; *Plp1*: 55 ± 4 for WT and 28 ± 3 for $CnB1^{\Delta Cnp1}$; Ki67: 100 ± 8.7 for WT and 93.7 ± 9.6 for $CnB1^{\Delta Cnp1}$; cleaved caspase 3: 100 ± 10.8 for WT and 82.9 ± 20.5 for $CnB1^{\Delta Cnp1}$). Statistical significance was determined by two-tailed Student's t-test (*, P ≤0.05; **, P ≤0.01; ***, P ≤0.001).

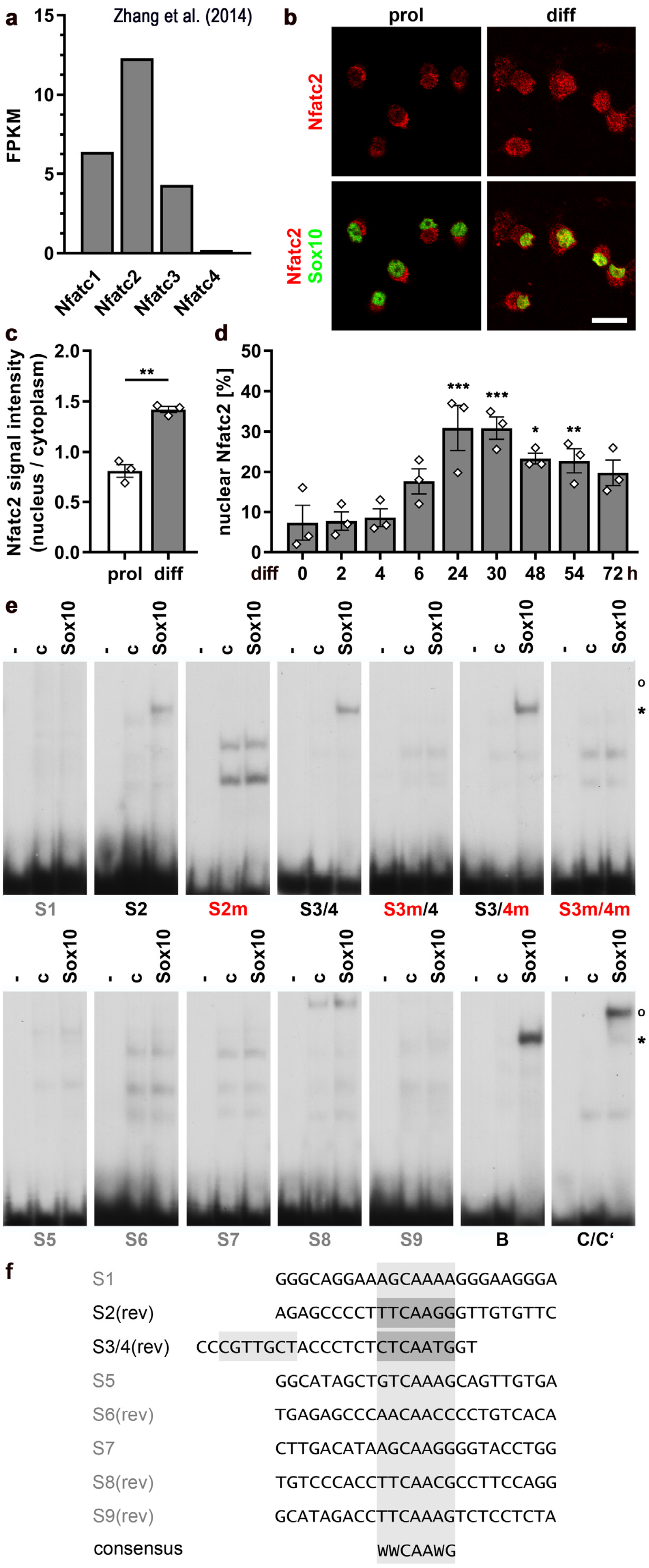

a
Zhang et al. (2014)
FPKM
15
10
5
0
Nfatc1
Nfatc2
Nfatc3
Nfatc4
b
prol
diff
Nfatc2
Nfatc2 Sox10
c
Nfatc2 signal intensity (nucleus / cytoplasm)
2.0
1.5
1.0
0.5
0.0
**
prol
diff
d
nuclear Nfatc2 [%]
50
40
30
20
10
0
***
***
*
**
diff 0 2 4 6 24 30 48 54 72 h
e
-
c
Sox10
o
*
S1
S2
S2m
S3/4
S3m/4
S3/4m
S3m/4m
S5
S6
S7
S8
S9
B
C/C'
f
S1 GGGCAGGAAAGCAAAAGGGAAGGGA
S2(rev) AGAGCCCCTTTCAAGGGTTGTGTTC
S3/4(rev) CCCGTTGCTACCCTCTCTCAATGGT
S5 GGCATAGCTGTCAAAGCAGTTGTGA
S6(rev) TGAGAGCCCAACAACCCCTGTCACA
S7 CTTGACATAAGCAAGGGGTACCTGG
S8(rev) TGTCCCACCTTCAACGCCTTCCAGG
S9(rev) GCATAGACCTTCAAAGTCTCCTCTA
consensus WWCAAWG

Supplementary Figure 3: Nfats are expressed in rodent oligodendrocytes. (a) Relative expression levels of *Nfat* mRNAs in mouse OPCs purified by immunopanning from mouse cortex. Values are given in FPKM and were taken from published RNASeq data (GSE52564) [22]. **(b)** Immunocytochemical staining of primary rat oligodendroglial cells that were cultured under proliferating conditions for one day (prol) or under differentiating conditions for 3 days (diff), with antibodies directed against Nfatc2 (red) and Sox10 (green). Scale bar, 20 µm. (**c**) Signal intensities for nuclear and cytoplasmic Nfatc2 were determined on confocal photographs of cultured oligodendroglial cells kept under proliferating (open bars) or under differentiating (grey bars) conditions after staining with antibodies directed against Nfatc2 and Sox10 using NIH image J software. Intensities were then put into relation. Three independent experiments were performed, with 30 cells counted per experiment. (Values: 0.81 ± 0.11 for prol and 1.42 ± 0.05 for diff). (**d**) Determination of the nuclear amount of a Nfatc2-YFP fusion protein in Oli-neu cells transfected with an expression plasmid for Nfatc2-YFP and H2B-mCherry before and 2-72 h after induction of differentiation with 1 mM dibutyryl-cAMP. Treatment started 1 day after transfection. Fluorescent signals for Nfatc2-YFP and H2B-mCherry were measured in single cells on photographs by NIH Image J software and the fraction of Nfatc2-YFP signal that overlapped with the nuclear mCherry signal was determined. Three independent experiments were performed, with at least 50 cells counted per condition. (Values: 6.8 ± 1.3 % before treatment; 7.8 ± 3.9 % after 2 h; 8.6 ± 3.8 % after 4 h; 17.8 ± 5.4 % after 6 h; 30.9 ± 4.8 % after 24 h; 30.9 ± 4.8 % after 30 h; 23.3 ± 2.3 % after 48 h; 22.8 ± 5.2 % after 54 h; 19.8 ± 5.6 % after 72 h). **(e)** EMSA with radiolabelled double-stranded oligonucleotides encompassing one or two closely spaced putative Sox10 binding sites from ECR87 in wildtype (S1 – S9, in grey or black) or mutant version (S2m, S3m/4, S3/4m, S3m/4m in red). Oligonucleotides were incubated in the absence (-), or presence (c, Sox10) of protein extracts before gel electrophoresis as indicated above the lanes. Extracts were from mock-transfected HEK293 cells (c) or HEK293 cells expressing full length Sox10 (Sox10). Oligonucleotides with site B and site C/C‘ from the promoter of the *Mpz* (myelin protein zero) gene served as positive control for Sox10 monomer and dimer binding. Positions of bands indicative of Sox10 binding are marked by circle for dimers and asterisk for monomers. **(f)** Sequences of the oligonucleotides used in EMSA. Oligonucleotides sequences were aligned with their potential (light grey) or verified (dark grey) Sox binding sites to the consensus. Where indicated, sequences are in reverse orientation (rev). Statistical significance was determined by Student's t test in (c) and Bonferroni-corrected two-way ANOVA in (d) (*, P ≤0.05; **, P ≤0.01; ***, P ≤0.001).

a

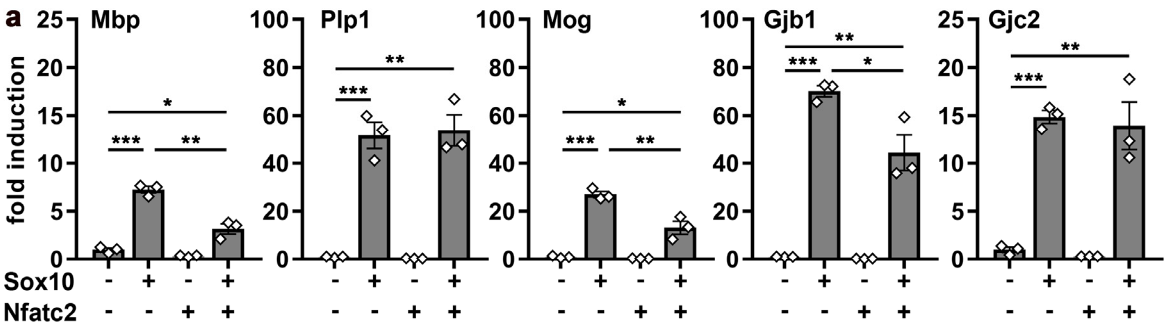

b

	Ascl1	Hes5	Id2	Id4	Klf6	Klf9	Myrf	Nkx2.2	Nkx6.2	Sip1	Sp7	Tcf7l2	Yy1	Zeb1	Zfp191
ECR	2	0	1	2	1	1	4	4	4	4	1	4	0	1	2
Sox10 peak	+4<br>+16		+62	+45<br>+54	-0.5	-120	-3<br>+8<br>+36<br>+97	-115<br>+5<br>+45	-46<br>+33<br>+46<br>+95	-3<br>+9<br>+50<br>+90	+10	-122<br>-70<br>+32<br>+109		+45	-1<br>-0.3
Olig2 peak			+62	+54				-19<br>+5<br>+45	-46<br>+33	+90					

c

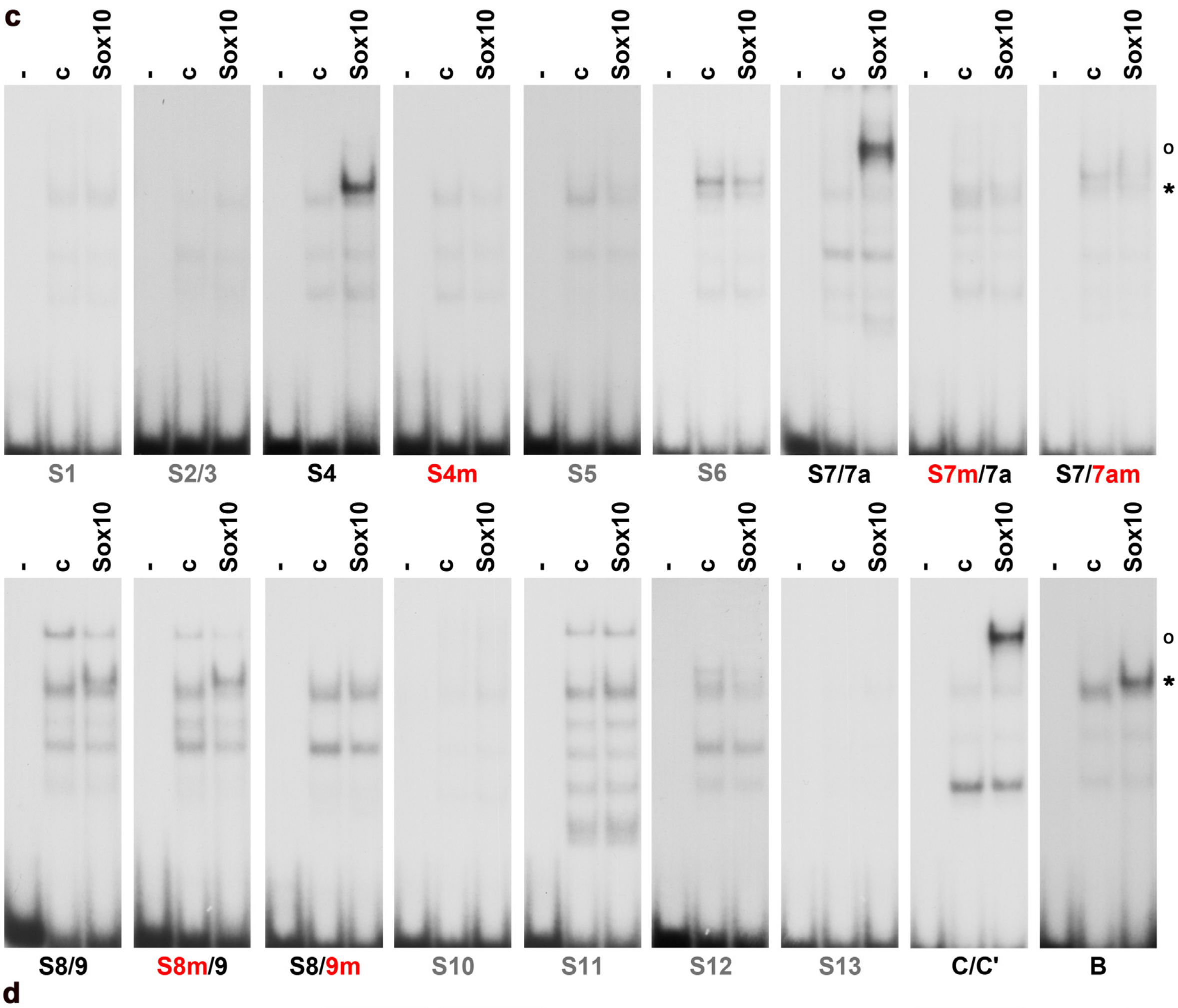

d

S1 ACGGGCATCTTCAAAAGTTCAAGAT
S2/3 CTGCTTCCATGTGAAAACCATGGTC
S4(rev) AAATACCACTTCATTGTCTGAGCCT
S5 TGGAGAATAAACAAACTTCTGAATG
S6(rev) TTGGATCAATATGATCGATGGCCAC
S7/7a(rev) TGTTTATTATTGGATCAATATGATC
S8/9(rev) ACTCCCATTATAATGCAGTGTTTAT
S10(rev) CTCATAGCATTCCAAGCAAGGGCTC
S11 AAAATCTTCCATTAATTCAGTGAAA
S12 TATATATCTATCAGAGACATGTAGT
S13 GAGACCAGGTTCACAGCAGTTTTCT
consensus WWCAAWG

Supplementary Figure 4: Sox10 activates myelin regulatory regions and binds to *Nkx2.2* ECR19 (a) Transient transfections of N2a cells were performed with luciferase reporters under control of regulatory regions from myelin genes in the absence (-) or presence (+) of Sox10 and constitutively active Nfatc2 as effectors (n = 3). An upstream *Mbp*, the WmN1 *Plp1* or an intronic *Mog* enhancer were used as myelin-specific regulatory regions in combination with a minimal promoter as well as the *Gjb1* and *Gjc2* promoter regions. Activation of reporter gene expression was determined in extracts 48 h post transfection and is presented as fold inductions ± SEM with transfections in the absence of effectors arbitrarily set to 1 for each regulatory region (*Mbp*: 7.3 ± 0.6 for Sox10, 0.3 ± 0.1 for Nfatc2 and 3.2 ± 0.9 for Sox10 in combination with Nfatc2; *Plp1*: 51.7 ± 9.5 for Sox10, 0.32 ± 0.1 for Nfatc2 and 53.8 ± 11.3 for Sox10 in combination with Nfatc2; *Mog*: 27.0 ± 2.2 for Sox10, 0.3 ± 0.1 for Nfatc2 and 13.2 ± 4.7 for Sox10 in combination with Nfatc2; *Gjb1*: 70.2 ± 3.9 for Sox10, 0.3 ± 0.1 for Nfatc2 and 44.4 ± 13.0 for Sox10 in combination with Nfatc2; *Gjc2*: 14.9 ± 1.1 for Sox10, 0.3 ± 0.0 for Nfatc2 and 13.9 ± 4.3 for Sox10 in combination with Nfatc2). Statistical significance was determined by two-tailed Student's t test (*, $P \leq 0.05$; **, $P \leq 0.01$; ***, $P \leq 0.001$). (**b**) List of ECRs in a 300 kb interval around 15 genes that code for oligodendroglial transcription factors and possess binding peaks for Sox10 and/or Olig2 in published ChIP-Seq studies (GSE64703 and GSE42447). Total number and positions in kb relative to the transcriptional start site are listed. **(c)** EMSA with radiolabelled double-stranded oligonucleotides encompassing one or two closely spaced putative Sox10 binding sites from ECR19 in wildtype (S1 – S13, in grey or black) or mutant version (S4m, S7m/7a, S7/7am, S8m/9 and S8/9m, in red). Oligonucleotides were incubated in the absence (-), or presence (c, Sox10) of protein extracts before gel electrophoresis as indicated above the lanes. Extracts were from mock-transfected HEK293 cells (c) or HEK293 cells expressing full length Sox10 (Sox10). Oligonucleotides with site B and site C/C' from the promoter of the *Mpz* (myelin protein zero) gene served as positive control for Sox10 monomer and dimer binding [50]. Positions of bands indicative of Sox10 binding are marked by circle for dimers and asterisk for monomers. **(d)** Sequences of the oligonucleotides used in EMSA. For further explanation see Suppl. Fig. 3f.

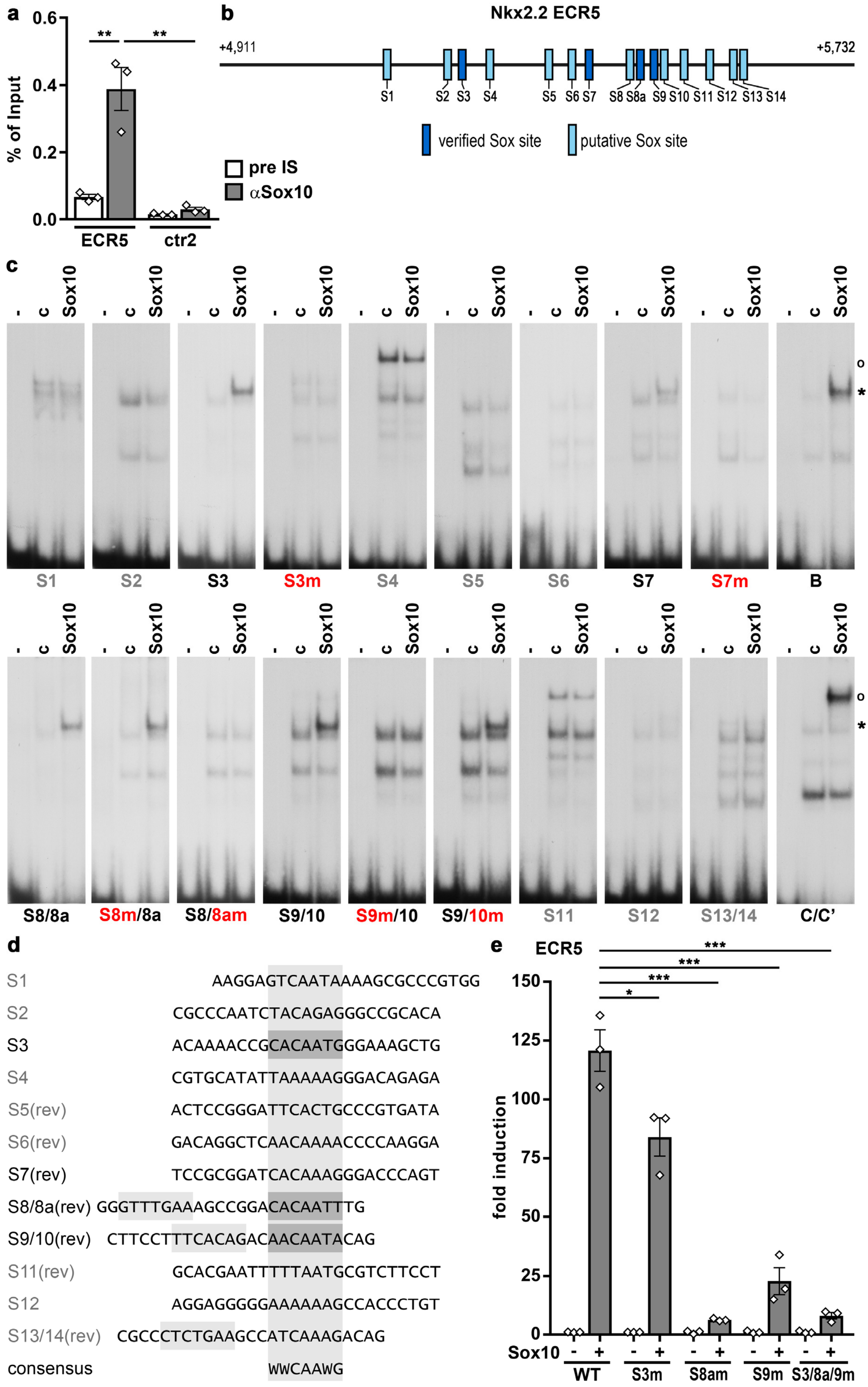
a
% of Input
0.6
0.4
0.2
0.0
**
ECR5
ctr2
pre IS
αSox10
b
Nkx2.2 ECR5
+4,911
+5,732
S1 S2 S3 S4 S5 S6 S7 S8 S8a S9 S10 S11 S12 S13 S14
verified Sox site
putative Sox site
c
- c Sox10
S1 S2 S3 S3m S4 S5 S6 S7 S7m B
S8/8a S8m/8a S8/8am S9/10 S9m/10 S9/10m S11 S12 S13/14 C/C'
o
*
d
S1 AAGGAGTCAATAAAAGCGCCCGTGG
S2 CGCCCAATCTACAGAGGGCCGCACA
S3 ACAAAACCGCACAATGGGAAAGCTG
S4 CGTGCATATTAAAAAGGGACAGAGA
S5(rev) ACTCCGGGATTCACTGCCCGTGATA
S6(rev) GACAGGCTCAACAAAACCCCAAGGA
S7(rev) TCCGCGGATCACAAAGGGACCCAGT
S8/8a(rev) GGGTTTGAAAGCCGGACACAATTTG
S9/10(rev) CTTCCTTTCACAGACAACAATACAG
S11(rev) GCACGAATTTTTAATGCGTCTTCCT
S12 AGGAGGGGGAAAAAAGCCACCCTGT
S13/14(rev) CGCCCTCTGAAGCCATCAAAGACAG
consensus WWCAAWG
e
ECR5
fold induction
150
125
100
75
50
25
0
***
*
Sox10
- +
WT S3m S8am S9m S3/8a/9m

Supplementary Figure 5: ECR5 represents a second Sox10-dependent enhancer of the *Nkx2.2* gene (a) ChIP on primary rat oligodendrocytes cultured *in vitro* for 4 days under differentiating conditions (n = 3) using rabbit pre-immune (pre IS, open bars) and anti-Sox10 (αSox10, grey bars) antiserum. Amounts of immunoprecipitated chromatin for ECR5 and control region (ctr2) were determined by qPCR and are presented as percent of input (control region: 0.014 ± 0.002 for pre IS and 0.030 ± 0.006 for αSox10; ECR5: 0.066 ± 0.008 for pre IS and 0.388 ± 0.064 for αSox10). **(b)** Localization of putative (light blue) and EMSA-confirmed (dark blue) binding sites for Sox10 in ECR5. Positions on left and right correspond to first and last basepairs of mouse ECR5 relative to the transcriptional start site. **(c)** EMSA with radiolabelled double-stranded oligonucleotides encompassing one or two closely spaced putative Sox10 binding sites from ECR5 in wildtype (S1 – S14, in grey or black) or mutant version (S3m, S7m, S8m/8a, S8/8am, S9m/10 and S9/10m, in red). Oligonucleotides were incubated in the absence (-), or presence (c, Sox10) of protein extracts before gel electrophoresis as indicated above the lanes. Extracts were from mock-transfected HEK293 cells (c) or HEK293 cells expressing full length Sox10 (Sox10). Oligonucleotides with site B and site C/C‘ from the promoter of the *Mpz* (myelin protein zero) gene served as positive control for Sox10 monomer and dimer binding. Positions of bands indicative of Sox10 binding are marked by circle for dimers and asterisk for monomers. **(d)** Sequences of the oligonucleotides used in EMSA. Oligonucleotides sequences were aligned with their potential (light grey) or verified (dark grey) Sox binding sites to the consensus. Where indicated, sequences are in reverse orientation (rev). **(e)** Transient transfections of N2a cells with ECR5 luciferase reporters in wildtype (WT) or mutant versions with inactivated Sox10 binding sites (S3m, S8am, S9m and S3/8a/9m) in the absence (-) or presence (+, grey bars) of Sox10 (n = 3). Sox10-dependent activations of the reporters were determined in extracts 48 h post transfection and are presented as fold inductions ± SEM (WT: 120.7 ± 8.8; S3m: 84.0 ± 8.1; S8am 6.3 ± 0.3; S9m 22.9 ± 5.7; S4/8a/9m 8.1 ± 1.4). Statistical significance in (a) and (e) was determined by two-tailed Student’s t-test (*, P ≤0.05; **, P ≤0.01; ***, P ≤0.001).

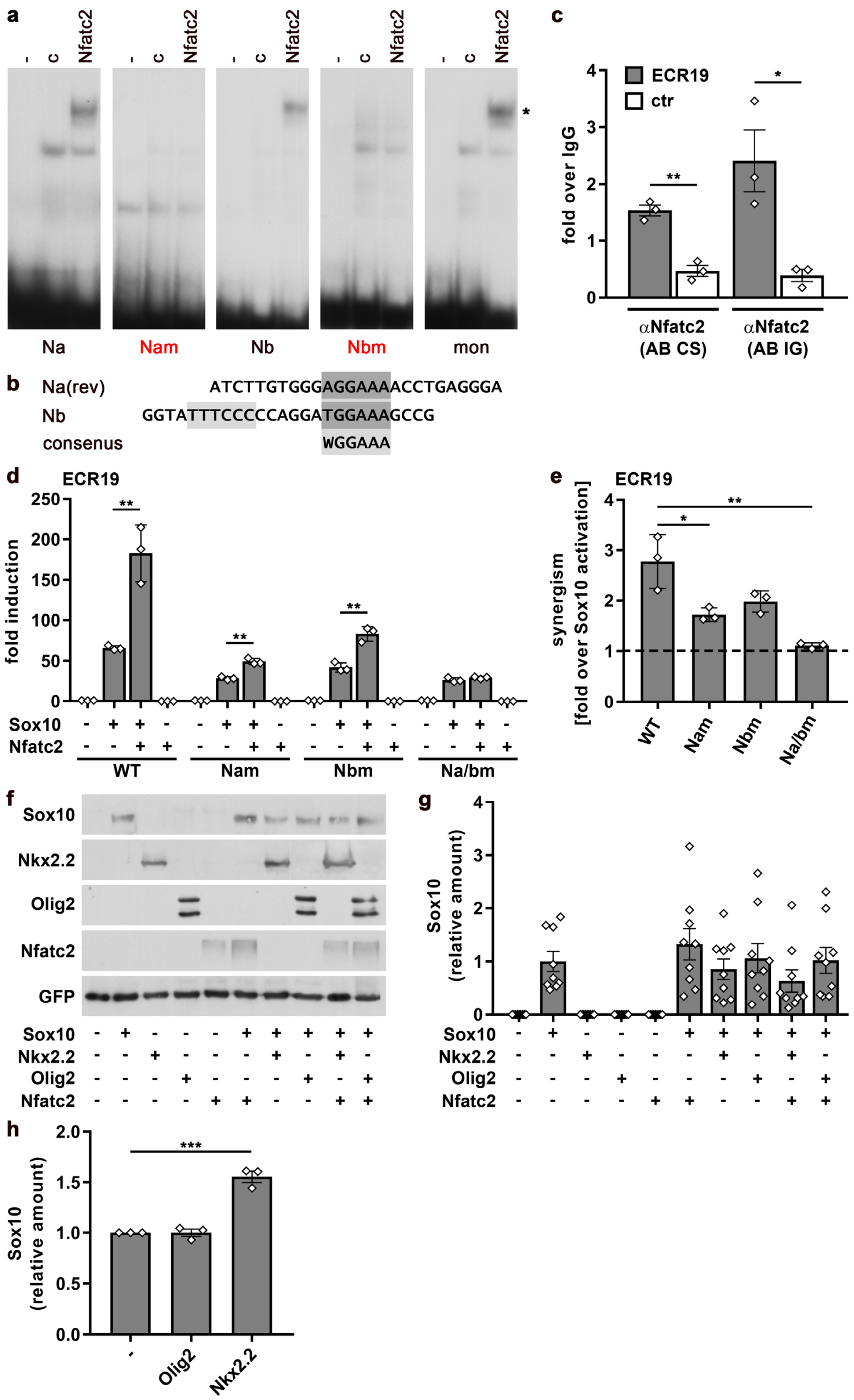

a
-
c
Nfatc2
*
Na
Nam
Nb
Nbm
mon
b
Na(rev) ATCTTGTGGGAGGAAAACCTGAGGGA
Nb GGTATTTCCCCCAGGATGGAAAGCCG
consenus WGGAAA
c
ECR19
ctr
fold over IgG
αNfatc2 (AB CS)
αNfatc2 (AB IG)
d
ECR19
fold induction
Sox10
Nfatc2
WT
Nam
Nbm
Na/bm
e
ECR19
synergism [fold over Sox10 activation]
WT
Nam
Nbm
Na/bm
f
Sox10
Nkx2.2
Olig2
Nfatc2
GFP
g
Sox10 (relative amount)
h
Sox10 (relative amount)
-
Olig2
Nkx2.2

Supplementary Figure 6: Nfatc2 has to bind ECR19 to cooperate with Sox10 during Nkx2.2 activation. (a) EMSA with radiolabelled double-stranded oligonucleotides encompassing the putative Nfat binding sites from ECR19 in wildtype (Na, Nb, in black) or mutant version (Nam, Nbm, in red). Oligonucleotides were incubated in the absence (-), or presence (c, Nfatc2) of protein extracts before gel electrophoresis as indicated above the lanes. Extracts were from mock-transfected HEK293 cells (c) or HEK293 cells expressing constitutively active Nfatc2 (Nfatc2). The position of Nfatc2-containing complexes is marked by asterisk. Mon, oligonucleotide with Nfat binding site from *Rag1* promoter. **(b)** Sequences of the oligonucleotides used in EMSA. Oligonucleotides sequences were aligned with their potential (light grey) or verified (dark grey) Nfat binding sites to the consensus. Where indicated, sequences are in reverse orientation (rev). **(c)** ChIP on primary rat oligodendrocytes cultured *in vitro* for one day under differentiating conditions (n = 3) using two different anti-Nfatc2 antibodies (AB CS and AB IG). Amounts of immunoprecipitated chromatin for ECR19 (grey bars) and control region (ctr, open bars) were determined by qPCR and are presented as fold over IgG (ECR19: 1.53 ± 0.10 and 2.41 ± 0.39 for Nfatc2; ctr: 0.47 ± 0.10 and 0.39 ± 0.11). **(d)** Transient transfections of N2a cells with ECR19 luciferase reporters in wildtype (WT) or mutant versions with inactivated Nfatc2 binding sites (Nam, Nbm, Na/bm) in the absence (-) or presence (+) of Sox10 and/or constitutively active Nfatc2 as effectors (n = 3). Activation of reporter gene expression was determined in extracts 48 h post transfection and is presented as fold inductions ± SEM over transfections without effector (WT: 65.8 ± 1.6 for Sox10, 0.1 ± 0.0 for Nfatc2 and 182.8 ± 20.3 for Sox10 and Nfatc2; Nam: 28.5 ± 1.2 for Sox10, 0.1 ± 0.0 for Nfatc2 and 49.1 ± 2.2 for Sox10 and Nfatc2; Nbm: 41.9 ± 3.3 for Sox10, 0.1 ± 0.0 for Nfatc2 and 83.0 ± 5.2 for Sox10 and Nfatc2; Na/bm: 26.4 ± 1.7 for Sox10, 0.1 ± 0.0 for Nfatc2 and 29.3 ± 0.8 for Sox10 and Nfatc2). **(e)** For better visualization of cooperativity between Sox10 and Nfatc2 on ECR19, fold inductions from (d) were normalized for each construct to values obtained in the presence of Sox10 (dotted horizontal line). (WT: 2.8 ± 0.3; Nam: 1.7 ± 0.1; Nbm: 2.0 ± 0.1; Na/bm: 1.1 ± 0.0). (**f,g**) Determination of protein amounts in N2a cells transiently transfected with expression plasmids for GFP and various combinations of Sox10, Nkx2.2, Olig2 and constitutively active Nfatc2 using Western blotting (f, see also Suppl. Fig. 8). From Western blots (n = 9), Sox10 and GFP amounts were quantified (g). After normalization to GFP, Sox10 amounts in N2a cells transfected with no other transcription factor were arbitrarily set to 1 (Sox10 amounts: 0 for cells transfected only with GFP or with combinations of GFP and Olig2, Nkx2.2 or Nfatc2; 1 ± 0.2 for cells transfected with GFP and Sox10; 1.3 ± 0.3 for cells transfected with GFP, Sox10 and Nfatc2; 0.9 ± 0.2 for cells transfected with GFP, Sox10 and Nkx2.2; 1.1 ± 0.3 for cells transfected with GFP, Sox10 and Olig2; 0.6 ± 0.2 for cells transfected with GFP, Sox10, Nkx2.2 and Nfatc2; 1 ± 0.2 for cells transfected with GFP, Sox10, Olig2 and Nfatc2). (**h**) Quantification of Sox10 amounts in primary oligodendroglial cultures 3 days after transduction with Olig2 or Nkx2.2 overexpressing retroviruses by Western blotting (n = 3). After normalization to Gapdh, Sox10 amounts in untransduced cultures were arbitrarily set to 1 (Sox10 amounts: 1 ± 0.1 in Olig2-overexpressing cultures; 1.6 ± 0.1 in Nkx2.2-overexpressing cultures). Statistical significance was determined by two-tailed Student's t-test in (c-e) and (h) and Bonferroni-corrected one-way ANOVA in (g) (*, P ≤0.05; **, P ≤0.01; ***, P ≤0.001).

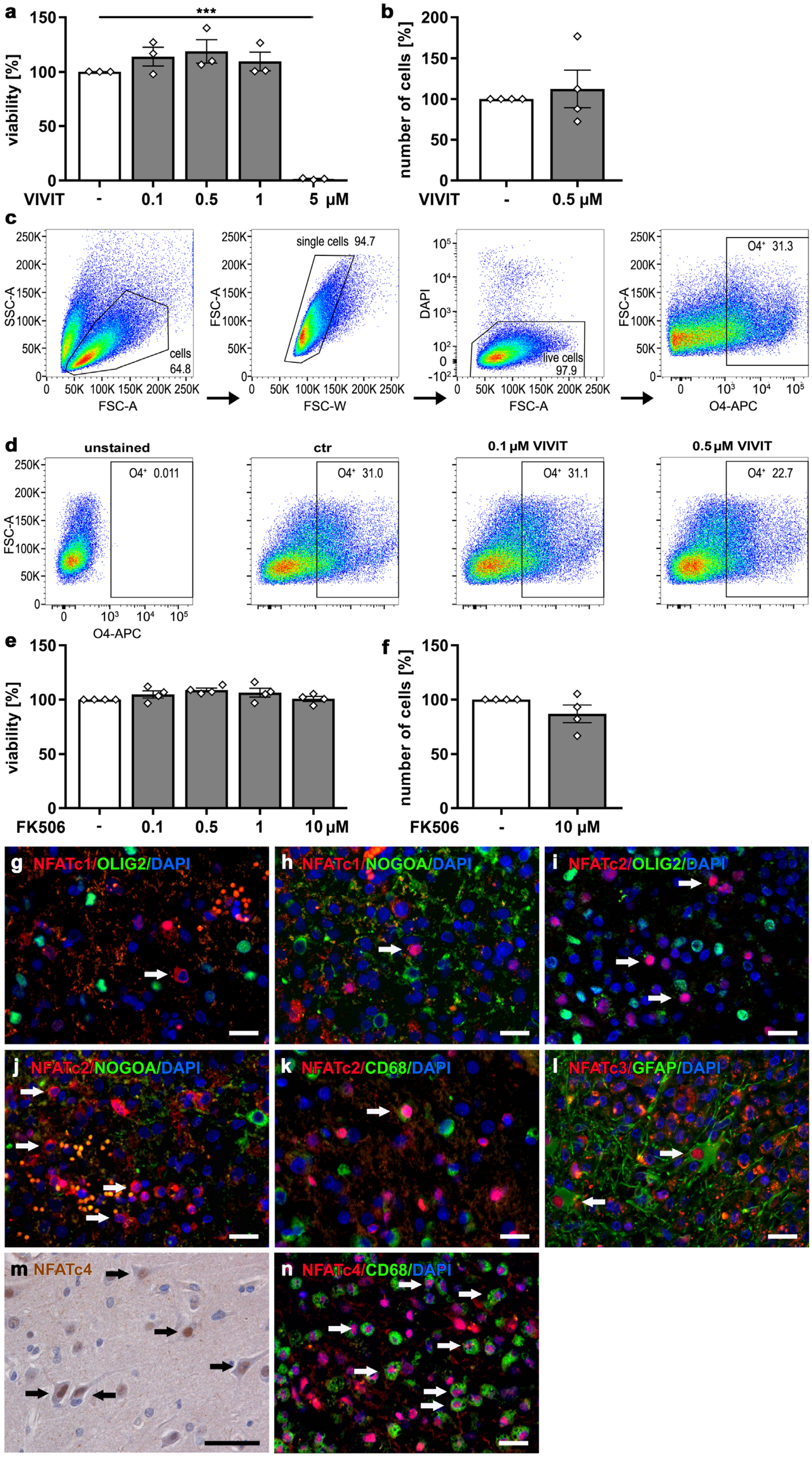

a
***
viability [%]
150
100
50
0
VIVIT
-
0.1
0.5
1
5 µM
b
number of cells [%]
200
150
100
50
0
VIVIT
-
0.5 µM
c
SSC-A
FSC-A
cells 64.8
FSC-W
single cells 94.7
DAPI
live cells 97.9
O4-APC
O4+ 31.3
d
unstained
O4+ 0.011
ctr
O4+ 31.0
0.1 µM VIVIT
O4+ 31.1
0.5 µM VIVIT
O4+ 22.7
e
viability [%]
FK506
-
0.1
0.5
1
10 µM
f
number of cells [%]
FK506
-
10 µM
g NFATc1/OLIG2/DAPI
h NFATc1/NOGOA/DAPI
i NFATc2/OLIG2/DAPI
j NFATc2/NOGOA/DAPI
k NFATc2/CD68/DAPI
l NFATc3/GFAP/DAPI
m NFATc4
n NFATc4/CD68/DAPI

Supplementary Figure 7: NFAT proteins are present in oligodendrocytes and other cell types of the human brain. (**a**) Viability of human iOL kept for 48 h in the absence (-, open bar) or presence (grey bars) of VIVIT at concentrations between 0.1 µM and 5 µM (n = 3). Viability in the absence of VIVIT was arbitrarily set to 100 percent and values were analysed in pairwise fashion (100 ± 0% in the absence of VIVIT; 114 ± 15% at 0.1 µM VIVIT; 119 ± 19% at 0.5 µM VIVIT; 109 ± 15% at 1 µM VIVIT; 2 ± 1% at 5 µM VIVIT). (**b**) Quantification of iOL numbers kept for 21 days under differentiating conditions in the absence (-, open bar) or presence of 0.5 µM VIVIT (grey bar) (n = 4). Numbers in the absence of VIVIT were arbitrarily set to 100 percent and values were analysed in pairwise fashion (100 ± 0% in the absence of VIVIT; 112 ± 46% at 0.5 µM VIVIT). (**c**) Representative flow cytometry analysis showing gating strategy to identify O4-positive cells. After initial FSC/SSC gating and doublet exclusion by a FSC-A vs FSC-W plot, live cells were gated as DAPI-negative cells. O4-positive cells were identified by using unstained cells and isotype controls. **(d)** Quantification of O4-positive iOL after 21 days under differentiating conditions in culture by FACS. Using the gating strategy shown in (c), cells were sorted after incubation with anti-O4 antibody. FACS blots show the percentage of O4-positive cells (boxed area) obtained in the absence (ctr) or presence of 0.1 µM or 0.5 µM VIVIT. Unstained cells served as control. (**e**) Viability of human iOL kept for 48 h in the absence (-, open bar) or presence (grey bars) of FK506 at concentrations between 1 µM and 10 µM (n = 4). Viability in the absence of FK506 was arbitrarily set to 100 percent and values were analysed in pairwise fashion (100 ± 0% in the absence of FK506; 105 ± 6% at 0.1 µM FK506; 109 ± 3% at 0.5 µM FK506; 106 ± 8% at 1 µM FK506; 101 ± 4% at 10 µM FK506). (**f**) Quantification of iOL numbers kept for 21 days under differentiating conditions in the absence (-, open bar) or presence of 10 µM FK506 (grey bar) (n = 4). Numbers in the absence of FK506 were arbitrarily set to 100 percent and values were analysed in pairwise fashion (100 ± 0% in the absence of VIVIT; 87 ± 16% at 10 µM FK506). (**g-n**) Antibodies directed against NFATc1 (red in g,h), NFATc2 (red in i-k), NFATc3 (red in l) and NFATc4 (brown in m, red in n) were combined in immunohistochemical stainings with antibodies directed against OLIG2 (green in g,i), NOGOA (green in h,j), CD68 (green in k,n) and GFAP (green in l). Scale bars, 50 µm. Arrows indicate NFATc1- or NFATc2-positive cells that do not express the oligodendroglial marker NOGOA or OLIG2 (g-j), NFATc2- and CD68-positive microglia/blood derived monocytes (k), NFATc3- and GFAP-positive astrocytes (l), NFATc4-positive neurons (m), and NFATc4- and CD68-positive microglia/blood derived monocytes (n). Statistical significance was determined by Bonferroni-corrected one-way ANOVA in (a) and (e) and two-tailed Student's t-test in (b) and (f) (*, $P \leq 0.05$; **, $P \leq 0.01$; ***, $P \leq 0.001$).

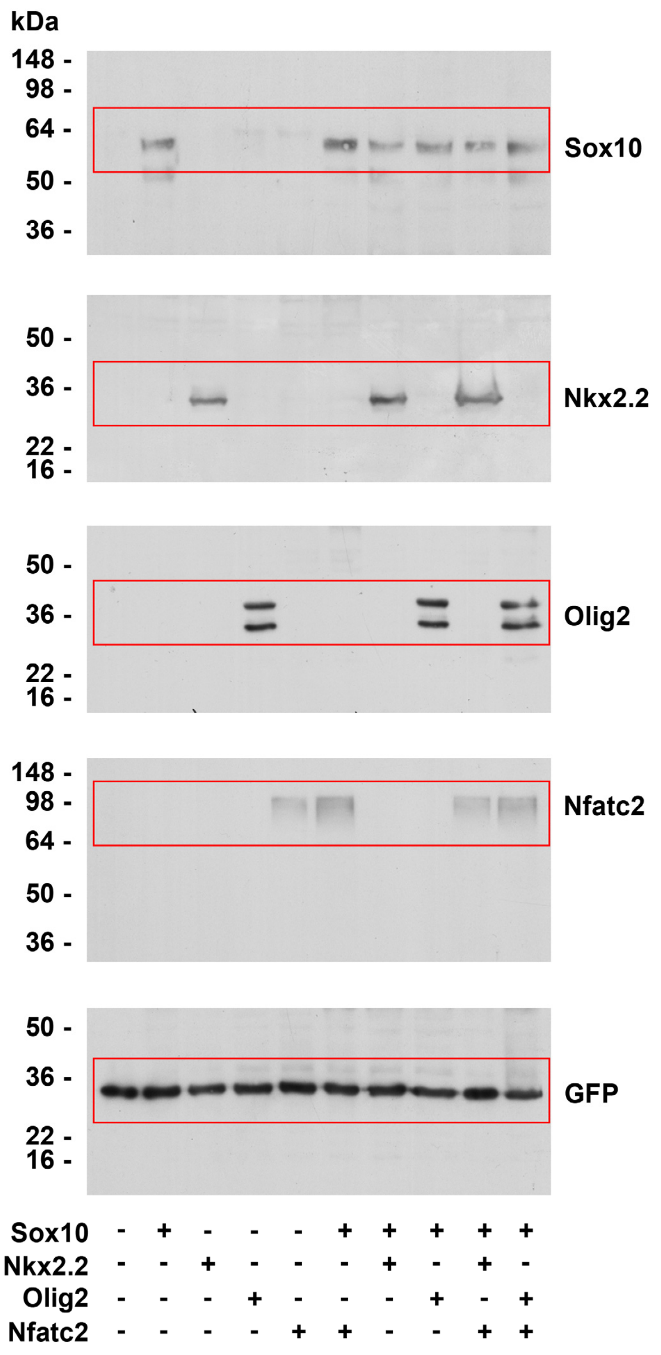

Supplementary Figure 8: Uncropped Western blot images of Supplementary Figure 6f. Regions shown in Suppl. Fig. 6f are boxed in red. Size markers in kilodalton (kDa) are shown on the left of each blot.
